# Transactions Third Annual Meeting of the Medical Society of Ga.

**Published:** 1852-10

**Authors:** 


*The Transactions of the Third Annual Meeting of the Medical Soeiety of the State of Georgia.*

We have received this interesting report; for this attention to us we return our thanks. We have not time to notice the contents particularly. They are, however, very creditable to the Medical Society of Georgia, and though we cannot hope that this or any other medical organization can effect much towards checking the fearful progress of empiricism or giving a healthy tone to the public mind upon the subject of medicine, we are glad to see that physicians are, notwithstanding all discouragements, earnestly at work to do their duty to this foolish and stiff-necked generation. Of the *true* physician the present world is not worthy.

